# Guillain–Barré syndrome post-SARS-CoV-2 vaccine: a systematic review and data analysis on its clinical, laboratory, electrophysiological, and radiological features

**DOI:** 10.3389/fneur.2024.1332364

**Published:** 2024-01-29

**Authors:** Kawther Hadhiah, Ali Alhashim, Hassan A. Al-Dandan, Eman Alhassan, Abdulaziz M. Alqarni, Abdullah Adil A. Memish, Majed Alabdali

**Affiliations:** Department of Neurology, College of Medicine, Imam Abdulrahman Bin Faisal University, Dammam, Saudi Arabia

**Keywords:** Guillain–Barré syndrome, cranial polyneuritis, acute sensory ataxia, acute inflammatory demyelinating polyneuropathy, acute motor axonal neuropathy, COVID-19 vaccine

## Abstract

**Introduction:**

Guillain–Barré syndrome (GBS) is a rare disease that affects almost 0.8–1.9 cases per 100,000 people worldwide every year. This is the most prevalent cause of subacute flaccid paralyzing illness today. It is a subacute inflammatory demyelinating polyradiculoneuropathy; the typical scenario involves ascending symmetrical flaccid paralysis, but in some circumstances, sensory, autonomic, and cranial neuropathy may also be involved. Several vaccines have been found to have complications since the previous century. Numerous case reports of GBS in the literature have been reported following COVID-19 vaccines in recent times.

**Objective:**

This study aimed to conduct a comprehensive examination of GBS cases that have been reported after COVID-19 vaccines; to analyze the descriptive statistical analysis of data gathered regarding clinical, laboratory, electrophysiological, and radiological characteristics; to discuss, based on the available evidence, whether the disease has a preference for a particular vaccine type; and to speculate on the potential pathogenesis.

**Methodology:**

This review has been carried out by recommendations of the Preferred Reporting Items for Systematic Reviews and Meta-Analyses (PRISMA) guidelines.

**Result:**

Reviewing 60 case reports illustrated that most of them are from the USA (18.1%) and the majority of affected individuals were males (60%). The results favored the association between vector-based SARS-CoV-2 vaccine, particularly AstraZeneca vaccine, and the GBS. The mean of symptoms onset is 11.4 days. The results of diagnostic tests such as LP are consistent mostly with albumin-cytological dissociation (81.81%), where brain and spine MRI was unremarkable in 59.52%. Regarding electrodiagnostic tests, AIDP is the most common variant (61.81%). The management was not consistent among the case reports. However, IVIG is the most frequent way of treating these patients (68.33%). The functional outcome was documented in 47 patients; 65% improved with medical management.

**Conclusion:**

This study aimed to conduct a systematic review of reported cases of GBS following COVID-19 vaccines and descriptive statistical analysis of collected data on clinical, laboratory, electrophysiological, and radiological features, to discuss, based on available results, whether the disease has a predilection to a specific vaccine type and to speculate the potential pathogenesis.

## Introduction

In December 2019, humanity recognized the first spark of the worst pandemic ever in the world by discovering a cluster of severe pneumonia cases in Wuhan city in China caused by severe acute respiratory coronavirus 2 (SARS-CoV-2). This has been rapidly disseminated all over the world in a short period leading to a global outbreak. Thus, the World Health Organization “WHO” in January 2020 declared the first pandemic to happen by SARS-CoV-2 (1).

Owing to the seriousness of the disease, the high contagiousness of the virus as well as lack of effective medication, a series of mandatory measures have been taken by governments worldwide, such as travel restrictions, case identification/tracking, and quarantine to prevent further spreading of the virus. However, due to the insufficiency and impracticality of the above measurements, there was a necessity for the development of an effective vaccine to control the disease spreading and to alleviate the severity of the disease. Hence, several vaccines with various mechanisms of action have been granted emergency use authorization (EUA) by the Food Drug Administration (FDA) as well as by the European Medicines Agency (EMA), namely Moderna and Pfizer-BioNTech, an mRNA-based vaccine, as well as Johnson & Johnson/Janssen, and AstraZeneca vaccine, a vector-based vaccine (2–5).

Despite the absence of serious adverse events during the conducted studies for COVID-19 vaccine approval (6, 7), WHO, FDA, and EMA advise close monitoring and reporting of any side effects that might occur after vaccination to US Vaccine Adverse Events Reporting System (VAERS) (8). This is because of deviation from regular approval guidelines due to the urgent need for authorization of COVID-19 vaccines as well as the newness of vaccination biotechnology. Subsequently, wide variety of side effects have been reported, ranging from local symptoms such as myalgia in injection site to mild systemic such as flu-like symptoms and fever to very serious conditions like hypercoagulability leading to pulmonary embolism or cerebral venous thrombosis “CVST” and autoimmune phenomena, for example, Guillain–Barré Syndrome (GBS) (9–13).

In this study, we are focusing on one of the most notable neurological side effects following immunization in adults, which is GBS as GBS has been a concern following all vaccines. GBS is an acute monophasic, rapidly progressive paralyzing illness due to inflammation of the nerve root and/or peripheral nerves (polyradiculoneuritis) usually provoked by a preceding infection or vaccination.

## Methodology

**Figure 1:**
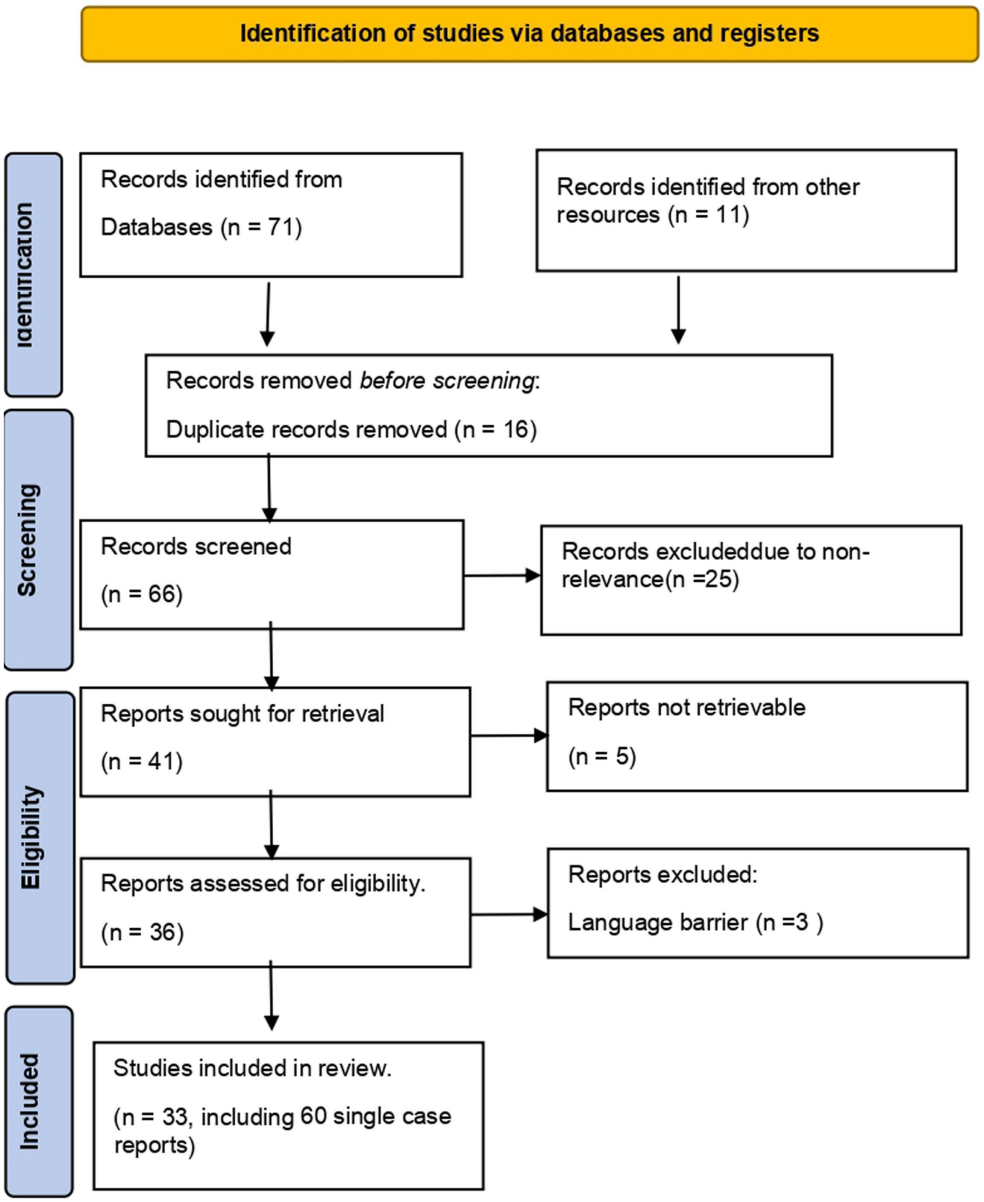
PRISMA flow chart of this study. This diagram shows the systematic process we followed to include articles reviewed by our search group through the year 2022.

This review has been carried out by recommendations of the Preferred Reporting Items for Systematic Reviews and Meta-analyses (PRISMA) guidelines (14). A comprehensive literature search on 14 January 2022 on PubMed was conducted for relevant published studies using the following keywords “Guillain–Barré syndrome,” “GBS,” “Miller Fisher syndrome,” “cranial polyneuritis,” “facial diplegia,” “Acute sensory ataxia,” “Bickerstaff encephalitis,” “acute inflammatory demyelinating polyneuropathy,” “acute motor axonal neuropathy” OR “acute motor and sensory axonal neuropathy” AND “COVID-19 Vaccine,” “Johnson & Johnson Vaccine COVID-19,” “Moderna Vaccine COVID-19,” “AstraZeneca Vaccine COVID-19,” “Vaxzevria Vaccine COVID-19,” “Ad26.COV2.S,” “Chadox1-s,” “mRNA-1273,” “BNT162b2,” OR “Pfizer Biotech vaccine COVID-19.” Reference lists of articles were comprehensively evaluated for relevance (see Figure 1).

Relevant articles that provided sufficient data according to a predefined list of 17 items (Table 1) were selected for final analysis. Furthermore, each selected article was further assessed manually for cross-references to find additional reports that may have been missed through the electronic search. Non-English articles and duplicated reports were excluded. The full text of eligible studies was retrieved and assessed, and the data were extracted according to a predefined list of 17 items (Table 1). A descriptive analysis of the data collected was performed using EXCEL. Continuous variables are presented as mean + standard deviation (SD), and categorical variables are reported as frequencies and percentages. Disagreements were resolved by team consensus.

**Table 1:** Characteristics of GBS post COVID vaccine.

Data	*N* (%)
Age	Mean 57.22 (20–86 years)
Sex	36 male (60%), 24 female (40%)
**Type of vaccine**	
Vector-based vaccine	40/60 (24 AZV, 4 J & JV) 66.6%
mRNA-based vaccine	19/60 (1 MV, 18 PBV) 31.6%
Other	1/60 1.6%
**Dose**	
1st	49 (81.6%)
2nd	2 (3.3%)
Uncertain	9 (15%)
Time interval between vaccine receipt and GBS onset	11.4 days (range 1–29 days)
**Country**	
USA	11 (18.3%)
UK	10 (16.6%)
India	10 (16.6%)
KR	2 (3.3%)
Malta	1 (1.6%)
Denmark	1 (1.6%)
Qatar	1 (1.6%)
Italy	2 (3.3%)
TR	1 (1.6%)
CA	3 (5%)
Brazil	1 (1.6%)
Tunisia	1 (1.6%)
Mexico	7 (11.6%)
Singapore	1 (1.6%)
Australia	5 (8.3%)
Japan	1 (1.6%)
Croatia	1 (1.6%)
Czech	1 (1.6%)
**Co-founders**	
Yes	8 (13.3%)
No	52 (86.6%)
**CSF analysis**	
Not done	7 (11.6%)
Done	53 (88.3%)
• Albumin-cytological dissociation	45/53 (84.9%)
• Mild pleocytosis (<25 cells/mm^3^)	4 (7.5%)
• Normal	4 (7.5%)
**MRI**	
Not done	18 (30%)
Done	42 (70%)
• Unremarkable MRI	25/42 (59.5%)
• Positive finding of cranial nerves or nerve root enhancement	17 (40.4%)
**NCS**	
Not done	5 (8.3%)
Done	55 (91.0%)
• AIDP	34/55 (61.8%)
• AMSAN	10 (18.1%)
• AMAN	9 (16.3%)
• Normal	2 (3.6%)
**Anti-ganglioside antibodies**	
Not reported/not done	37 (61.6%)
Done	23 (38.3%)
• Negative	22/23 (95.6%)
• Positive	1 (4.3%)
**COVID-19 PCR**	
Not done	27 (45%)
Done	33 (55%)
• Negative	33/ 33 (100%)
• Positive	0 (0%)
**Clinical phenotype (classification)**	
Classical GBS	38 (63.3%)
Pharyngeal-cervical-brachial weakness	0 (0%)
Paraparetic GBS	5 (8.3%)
Bifacial weakness with paraesthesias	9 (15%)
Pharyngeal (Bulbar) weakness	1 (1.6%)
MFS	4 (6.6%)
Sensory GBS	3 (5%)
**Clinical features**	
1-Prodrame (backache, headache, and leg pain)	22 (36.6%)
2-Facial palsy	30 (50%)
3-Bulbar palsy	14 (23.3%)
4-Tetraparesis/-plagia	27 (45%)
5-Paraparesis/-plagia	17 (28.3%)
Hyporeflexia/areflexia	46 (76.6%)
Sensory deficit	44 (73.3%)
Dysautonomia	12 (20%)
Respiratory failure	5 (8.3%)
Ophthalmoplegia	4 (6.6%)
**Brighton criteria**	
Level 1	25 (41.6%)
Level 2	19 (31.6%)
Level 3	2 (3.3%)
Level 4	14 (23.3%)
**Immunotherapy**	
IVIG	41 (68.3%)
PE	0 (0%)
Oral steroid	2 (3.3%)
IVIG and PE	5 (8.3%)
IVIG and oral steroid	4 (6.6%)
Symptomatic management	6 (10%)
None	2 (3.3%)
**Follow-up**	
Not documented	13 (21.6%)
Not improve	8 (17%)
Improve	39 (82.9%)
- Slightly improve	16 (41%)
- Significantly improve	20 (51.2%)
- Recover	3 (7.6%)
Death	(0%)

## Result

Thirty-three articles (60 single case reports) describing GBS variants and subtypes in patients’ post-SARS-CoV-2 vaccination were found. Sixteen duplicated reports were identified, and overall, 60 patients were in included the systematic review.

The demographic data, and the clinical, laboratory, and imaging findings of the 60 patients, are summarized in Table 1. Because of the characteristics of the reports, the low number of patients, and the variability in the reported features, we described the studies and summarized their results qualitatively and quantitatively, rather than by a meta-analysis approach.

The median age was 57.22 years, and the majority of patients were men (36/60; 60%). Overall, patients were reported from 18 countries but mostly from the USA (18.3%), UK (16.6%), and India (16.6%). Most patients have no other feasible triggers for GBS other than the COVID vaccine except in 8 patients among 60 patients (13.3%), who have been already diagnosed with autoimmune diseases, such as celiac disease and ulcerative colitis, or metabolic diseases like diabetes, end-stage renal disease, and hyperthyroidism. The reported cases mostly were after receiving vector-based vaccine 40/60 (66.6%), mainly related to AstraZeneca. Furthermore, the greater number of cases were after the first dose 49/60 (81.6%). The median time interval between vaccine receiving and GBS onset was 11.4 days (range 1–29 days).

Regarding GBS workup, CSF examination was performed in 53 (88.3%) patients and revealed a classical finding with albumin-cytological dissociation in 45/53 (84.9%). Nevertheless, there are mild pleocytosis (<25 cells/ mm3) in 4/53 (7.5%) patients and normal results in 4/53 (7.5%). Brain and spine MRI were taken for 42 (70%) patients. Most of them (25 patients) had unremarkable scans. However, the remaining 17 patients had either nerve root enhancement or cranial nerve enhancement. Most of the patients underwent nerve conduction studies (91.66%). Among them, 34 patients had acute inflammatory demyelinating polyradiculoneuropathy subtype (AIDP), 10 patients had the AMSAN subtype, and 9 patients had the AMAN subtype. Anti-ganglioside antibodies test was not reported/done for 37 patients (61.6%). However, 23 (38.2%) underwent the test where 22 (95.65%) had negative results and only one patient (4.3%) had positive results. COVID PCR test was done for 33 patients (55%), and all of them had negative results. On the other hand, it was not carried out for 27 patients (45%).

The COVID-19 vaccine-related GBS had variable clinical phenotypes as follows; 38 (63.3%) of them had classical GBS phenotype. Bifacial weakness with paraesthesia phenotype was seen in nine (15%). Paraparetic GBS phenotype occurred in five patients (8.3%). MFS was reported in four cases (6.5%). Isolated sensory GBS happened in three patients (5%). Finally, pharyngeal (Bulbar) weakness was present in one patient only (1.6%).

Regarding symptoms review, prodromal symptoms in terms of headache and backache were reported in 36.6%. Furthermore, 73.3% of the patients complained of sensory disturbance with or before stating the motor manifestation. Motor signs were noticed as facial palsy (50%), bulbar symptoms were found in 14 patients, and 8.3% (5 cases) of them progressed to respiratory failure and required intubation. Either tetraparesis or paraparesis found in 44 patients was 73.3%. Other motor dysfunction features like hyporeflexia or areflexia existed in the majority of the patients (76.6%). Dysautonomia was found in 12 patients (20%).

The Brighton diagnostic criteria of GBS were applied for the 60 patients: 25 (41.6%) reached level 1, 19 (31.6%) had level 2, 14 (23.3%) had level 4, and 2 had level 3 (3.3%).

The greatest number of patients were treated with IVIG 41/60 (68.3%), and 5 (8.33%) patients were treated with both IVIG and PE. However, none was treated with plasma exchange alone. The oral steroid was given to 2 (3.3%) and 4 (6.6%) patients who received combination therapy of oral steroids and IVIG. Two (3.3%) patients were not documented to receive any treatment, and six (10%) managed with symptomatic measures like gabapentin. As follow-up is only documented in 47 patients, 82.9% (39/47) of patients improved on the above therapies as follows: 16/39 (41%) with slight improvement, 20/39 (51.2%) with definitive improvement, and 3/39 (7.6%) patients recovered totally. No improvement was documented in 8 patients out of 47 patients who had follow-up (17%). Follow-up status was not documented in 13/60 (21.6%). No death was reported.

## Discussion

Guillain–Barré syndrome (GBS) is a rare disease that almost affects 0.8–1.9 cases per 100,000 people every year worldwide (15). It is a subacute inflammatory demyelinating polyradiculoneuropathy that presents typically with ascending symmetrical flaccid paralysis, however to a variable degree in some instances; it is combined with sensory, autonomic, and cranial neuropathy. The two most common forms of GBS are acute inflammatory demyelinating polyradiculoneuropathy (AIDP) and acute motor axonal neuropathy (AMAN). In addition, there are other several variants, including acute motor-sensory axonal GBS (AMSAN), pure sensory GBS, Miller Fisher syndrome, paraparetic GBS, pharyngeal-cervical-brachial GBS, bilateral facial palsy with paresthesia (BFP), and Bickerstaff brainstem encephalitis (BBE) (15). Most patients in our study (61.81%) had the classical clinical presentation, but virtually, all GBS variants and subtypes were reported.

In general, two-thirds of GBS cases are preceded by systematic infection 2–3 weeks before the onset. As per case-control studies, the most common causative organisms where antecedent infections have been documented are *Campylobacter jejuni*, cytomegalovirus, hepatitis E virus, *Mycoplasma pneumoniae*, Epstein–Barr, and Zika virus (16–18). Furthermore, vaccination has been attributed as an etiological cause of GBS. In 1976, the first putative correlation was with the swine flu vaccine in which a total of 532 patients had recently received vaccination before their onset of GBS (19). Afterward, the seasonal influenza vaccine showed a similar association as it was approved by a meta-analysis published in 2015 that concluded that the overall relative risk was 1.41 (95% CI, 1.20–1.66). Pandemic vaccines presented a higher risk (RR = 1.84; 95% CI, 1.36–2.50) than seasonal vaccines (RR = 1.22; 95% CI, 1.01–1.48) (20).

Recently, COVID-19 infection has been identified to be associated with central and peripheral nervous system involvement during the outbreak pandemic 2020. Systematic reviews as well as a meta-analysis based on 11 cohorts found an increased risk of sub-acute inflammatory demyelinating polyradiculoneuropathy in comparison with non-infected patients (21–23). Conversely, the causal relationship between COVID-19 vaccines and GBS is still under hot debate since the surge of reported cases of GBS after receiving the COVID-19 vaccine (Table 1). No GBS occurred in clinical trials of COVID-19 vaccines, except for one among 19,630 Ad26.COV’S recipients. However, a recent surveillance study of GBS after receipt of COVID-19 vaccines through the US Vaccine Adverse Event Reporting System (VAERS) suggested a potential small but statistically significant safety concern for Guillain–Barré syndrome following receipt of the Ad26.COV2. S vaccine, a vector-based vaccine. However, the findings are subject to the limitations of passive reporting systems and presumptive case definitions (24). Our findings of the study support that suspicion and propose a possible association between the vector-based vaccine, namely ChAdOx1 vaccine, and GBS; however, due to limitations and methodologies, it is impossible to confirm any causality and a large-scale epidemiological study is needed for that.

The pathogenesis of GBS is not fully understood, but it was attributed to molecular mimicry that will trigger humoral immune response, in which antibodies attack either the myelin membrane or the axon leading to the clinical phenomena of GBS (25–28). Similarly, the exact mechanism of SARS-CoV-2 vaccine-induced GBS is inconclusive (29). It was hypothesized to be a result of autoimmunity mediated by vaccine epitopes and the formation of host antibodies that cross-react with peripheral myelin proteins (30, 31). Reporting similar syndrome during the SARS-CoV-2 pandemic raised the possibility of molecular mimicry when SARS-CoV-2 spike protein binds to gangliosides on the cell surface of peripheral nerves and leads to autoantibodies formation and ends with myelin sheath and Schwann cell damage (29, 31). Other mechanisms such as humeral or T cell-mediated antibodies attacking peripheral nerve gangliosides leading to other GBS types are also postulated (29).

## Limitations

This article reviews case report studies retrospectively that were published at a certain time (September 2021 to January 2022), which might be affected by omitting other similar cases that did not exist as an article during that given period; furthermore, not all the reviewed cases were similar in diagnostic and therapeutic approach. A few of the included studies did not clarify clearly about rolling out other possible causative agents (e.g., infections). The short period of following some of those patients was not enough to judge the outcomes and recovery.

## Recommendations

The noticeable relation between a specific COVID vaccine type and GBS has to be highly concerned and accordingly a guideline for the appropriate selection of appropriate vaccines for those at high risk.

## Conclusion

We proposed a possible association between GBS and the ChAdOx1 vaccine. Nevertheless, further well-designed large-scale epidemiological studies are needed to confirm the potential association as well as laboratory research to find the exact pathogenesis.

## Author contributions

KH: Conceptualization, Supervision, Writing – review & editing. AlA: Conceptualization, Formal analysis, Supervision, Writing – review & editing. HA-D: Data curation, Validation, Writing – review & editing. EA: Conceptualization, Investigation, Writing – review & editing. AbA: Conceptualization, Investigation, Writing – review & editing. AM: Conceptualization, Data curation, Writing – review & editing. MA: Investigation, Supervision, Validation, Writing – review & editing.
